# Cognitive Style and Mobile E-Learning in Emergent Otorhinolaryngology-Head and Neck Surgery Disorders for Millennial Undergraduate Medical Students: Randomized Controlled Trial

**DOI:** 10.2196/jmir.8987

**Published:** 2018-02-13

**Authors:** Li-Ang Lee, Yi-Ping Chao, Chung-Guei Huang, Ji-Tseng Fang, Shu-Ling Wang, Cheng-Keng Chuang, Chung-Jan Kang, Li-Jen Hsin, Wan-Ni Lin, Tuan-Jen Fang, Hsueh-Yu Li

**Affiliations:** ^1^ Faculty of Medicine Chang Gung University Taoyuan Taiwan; ^2^ Graduate Institute of Clinical Medicine Sciences Chang Gung University Taoyuan Taiwan; ^3^ Department of Otorhinolaryngology-Head and Neck Surgery Linkou Chang Gung Memorial Hospital Taoyuan Taiwan; ^4^ Department of Computer Science and Information Engineering Graduate Institute of Medical Mechatronics Chang Gung University Taoyuan Taiwan; ^5^ Department of Neurology Linkou Chang Gung Memorial Hospital Taoyuan Taiwan; ^6^ Department of Laboratory Medicine Linkou Chang Gung Memorial Hospital Taoyuan Taiwan; ^7^ Department of Medical Biotechnology and Laboratory Science Graduate Institute of Biomedical Sciences Chang-Gung University Taoyuan Taiwan; ^8^ Department of Nephrology Linkou Chang Gung Memorial Hospital Taoyuan Taiwan; ^9^ Graduate Institute of Digital Learning and Education National Taiwan University of Science and Technology Taipei Taiwan; ^10^ Department of Surgery Linkou Chang Gung Memorial Hospital Taoyuan Taiwan

**Keywords:** cognitive style, e-learning, mobile technology, randomized controlled trial

## Abstract

**Background:**

Electronic learning (e-learning) through mobile technology represents a novel way to teach emergent otorhinolaryngology-head and neck surgery (ORL-HNS) disorders to undergraduate medical students. Whether a cognitive style of education combined with learning modules can impact learning outcomes and satisfaction in millennial medical students is unknown.

**Objective:**

The aim of this study was to assess the impact of cognitive styles and learning modules using mobile e-learning on knowledge gain, competence gain, and satisfaction for emergent ORL-HNS disorders.

**Methods:**

This randomized controlled trial included 60 undergraduate medical students who were novices in ORL-HNS at an academic teaching hospital. The cognitive style of the participants was assessed using the group embedded figures test. The students were randomly assigned (1:1) to a novel interactive multimedia (IM) group and conventional Microsoft PowerPoint show (PPS) group matched by age, sex, and cognitive style. The content for the gamified IM module was derived from and corresponded to the textbook-based learning material of the PPS module (video lectures). The participants were unblinded and used fully automated courseware containing the IM or PPS module on a 7-inch tablet for 100 min. Knowledge and competence were assessed using multiple-choice questions and multimedia situation tests, respectively. Each participant also rated their global satisfaction.

**Results:**

All of the participants (median age 23 years, range 22-26 years; 36 males and 24 females) received the intended intervention after randomization. Overall, the participants had significant gains in knowledge (median 50%, interquartile range [IQR]=17%-80%, *P*<.001) and competence (median 13%, IQR=0%-33%, *P*=.006). There were no significant differences in knowledge gain (40%, IQR=13%-76% vs 60%, IQR=20%-100%, *P*=.42) and competence gain (0%, IQR= −21% to 38% vs 25%, IQR=0%-33%, *P*=.16) between the IM and PPS groups. However, the IM group had a higher satisfaction score (8, IQR=6-9 vs 6, IQR=4-7, *P*=.01) compared with the PPS group. Using Friedman’s two-way nonparametric analysis of variance, cognitive styles (field-independent, field-intermediate, or field-dependent classification) and learning modules (IM or PPS) had significant effects on both knowledge gain (both adjusted *P*<.001) and satisfaction (both adjusted *P*<.001).

**Conclusions:**

Mobile e-learning is an effective modality to improve knowledge of emergent ORL-HNS in millennial undergraduate medical students. Our findings suggest the necessity of developing various modules for undergraduate medical students with different cognitive styles.

**Trial Registration:**

Clinicaltrials.gov NCT02971735; https://clinicaltrials.gov/ct2/show/NCT02971735 (Archived by WebCite at http://www.webcitation.org/6waoOpCEV)

## Introduction

### e-Learning Can Provide an Opportunity for Active Self-Directed Learning

The large investment involved in undergraduate medical education (UME) for students and society has led medical schools worldwide to seek strategies and methods to improve their students’ progress [1-3]. The cost of medical school tuition continues to increase annually [4], and medical students face substantial financial stress in the United States and Taiwan [5]. A reduction in medical training time has been shown to reduce medical school tuition fees [6]. Innovative curricula, quality of teaching, and primary care education are three major issues in UME [4,7], and novel UME should empower undergraduate medical students to use different learning strategies and learn outside the classroom by promoting self-directed learning [8]. For instance, electronic learning (e-learning) can provide an opportunity for active self-directed learning and the dissemination of knowledge in an interactive fashion [9].

### Cognitive Style Is Underresearched in the Context of Medical Education

Certain learning characteristics have been positively correlated with academic success; for instance, strong motivation and enjoying studying have been identified as positive predictors during the undergraduate year of medical school [10]. However, it has also been suggested that the predictive power of learning strategies such as cognitive style is underresearched in the context of medical education [11]. At least some undergraduate medical student–initiated learning situations have been reported to be consistent with their individual cognitive and instructional preferences [12]. The Group Embedded Figure Test (GEFT) was first applied to assess cognitive style and instructional materials for medical students in 1981 [13]. Field-independent (FI) learners have been shown to prefer and have better performance in problem-based learning and computer-assisted learning [14], and the preinstructional determination of cognitive style may help to select suitable instructional materials for these students while providing other instructional tools for field-dependent (FD) learners. However, when teaching millennials (also known as digital natives) who are paradoxically motivated by self-interest, the current understanding of these students may be inaccurate because of the considerable diversity in background, personality, and learning preference [15,16]. Therefore, educational reforms such as competency-based medical education have been implemented to better suit the current millennial generation of undergraduate medical students [17].

### Mobile Technology in e-Learning Has Gained Popularity

Mobile technology has gained popularity in recent years as a means of immediate interactive multimedia (IM) communication and to access the Internet. Embedded e-learning in a smartphone or tablet can affect the educational environment. In this context, it has been termed “mobile technology in e-learning (M-TEL),” and it has been reported to represent the next natural frontier in the evolution of e-learning [18]. Almost all e-learning today can be accessed from mobile devices, including medical education [19], patient education [20], and the development of mobile medical educators [21]. However, clinical teachers looking to use M-TEL therefore need to ensure that it will meet both the needs of their millennial learners and the requirements of the program.

### Students Need to Spend More of Their Time Outside the Classroom to Learn Otorhinolaryngology-Head and Neck Surgery

Reducing training time can limit the number of topics taught in an UME curriculum, including otorhinolaryngology-head and neck surgery (ORL-HNS). However, at least 20% of primary care complaints are related to ORL-HNS, and a substantial downstream effect on managing ORL-HNS problems has been reported in family medical practice [22]. In Taiwan, a reduction of approximate 20% in classroom lectures in 6-year medical programs was implemented in 2013. Therefore, the students need to be encouraged to spend more of their time outside the classroom to learn. In our pilot study [23], we found that M-TEL using IM modules could be an effective and satisfactory way to learn about emergent ORL-HNS disorders. In this study, we hypothesized that FI learners would prefer M-TEL technology compared with FD learners, and that they would have a better performance with a novel IM module (cases) compared with a conventional Microsoft PowerPoint show (PPS) module (controls). The control group also received identical instructional materials using the same mobile device.

## Methods

### Study Design

We conducted this prospective study from August 1, 2015 to July 31, 2017 at a university (Department of ORL-HNS, Faculty of Medicine, Chang Gung University, Taoyuan, Taiwan). This study included two parts: (1) pilot system-design study, and (2) validation study. This study was approved by the institutional review board of Chang Gung Medical Foundation (No: 105-5290C), and all procedures were conducted in compliance with the Declaration of Helsinki 1975. The participants were informed about the aims of the study, and written informed consent was obtained from all participants. The study proposal was registered at ClinicalTrials.gov (NCT02971735).

### Setting

**Figure 1:**
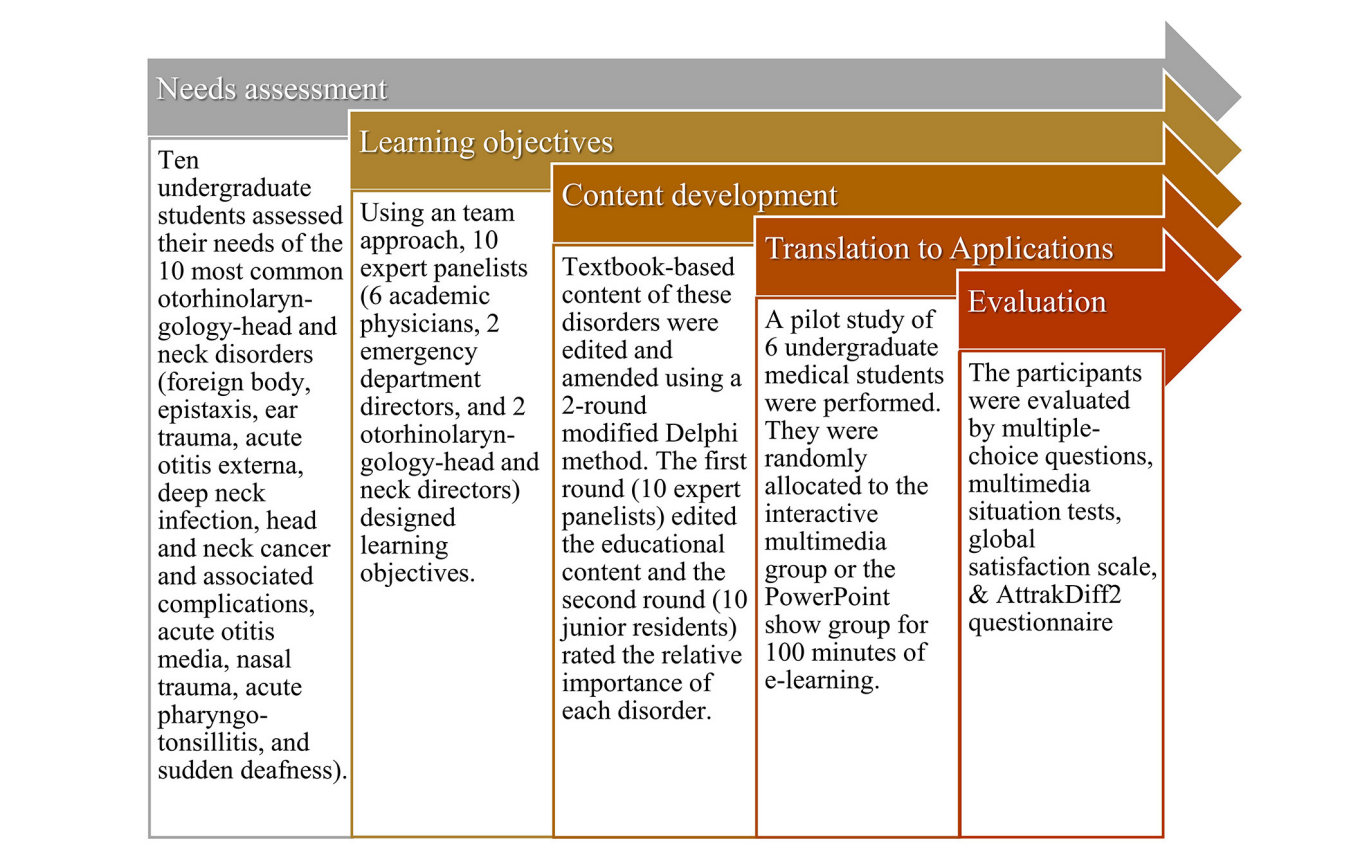
Analysis, design, development, implementation, and evaluation (ADDIE) model for designing effective instruction of mobile technology in electronic learning (e-learning).

**Figure 2:**
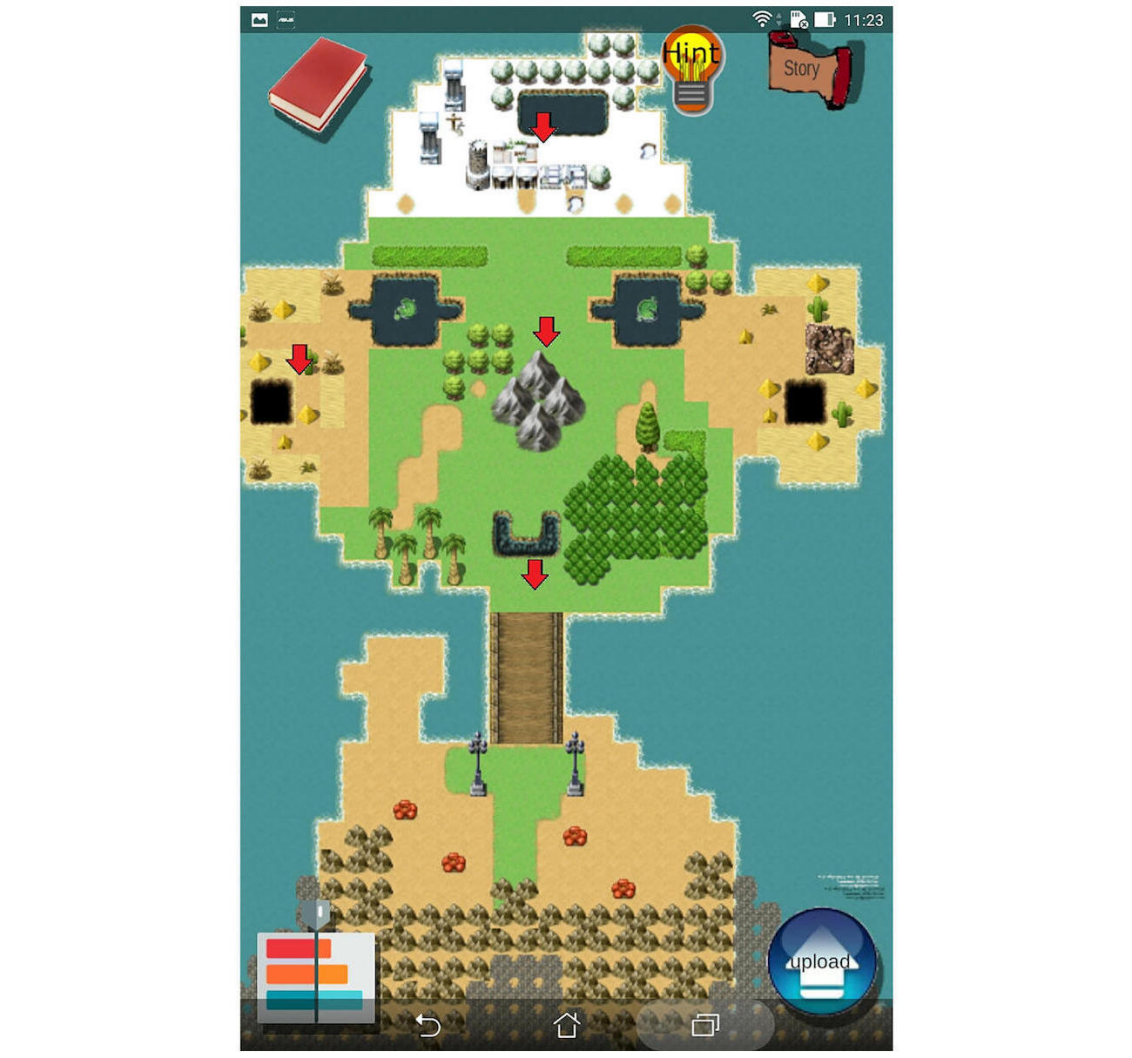
Start of the apps. Learners read the adventure story and objectives (story symbol), played four instructional domains (red arrow symbol), reviewed instructional materials (book symbol), assessed learning progress (bar chart symbol), and got the helps (hint symbol) on the start screen.

In the pilot system-design study [23], we established the instructional materials, including essential knowledge and competence of the 10 most common emergent ORL-HNS disorders using the analysis, design, development, implementation, and evaluation models [24] to design effective instruction for e-learning (Figure 1). All of the materials were developed according to the results of needs assessment in a focus group of undergraduate students and revised using a two-round modified Delphi method to develop the instructional content and assess the relative importance of each item. Storyboards and courseware of the IM and PPS modules were developed using the same user interface (Figure 2).

#### The Novel IM Module

**Figure 3:**
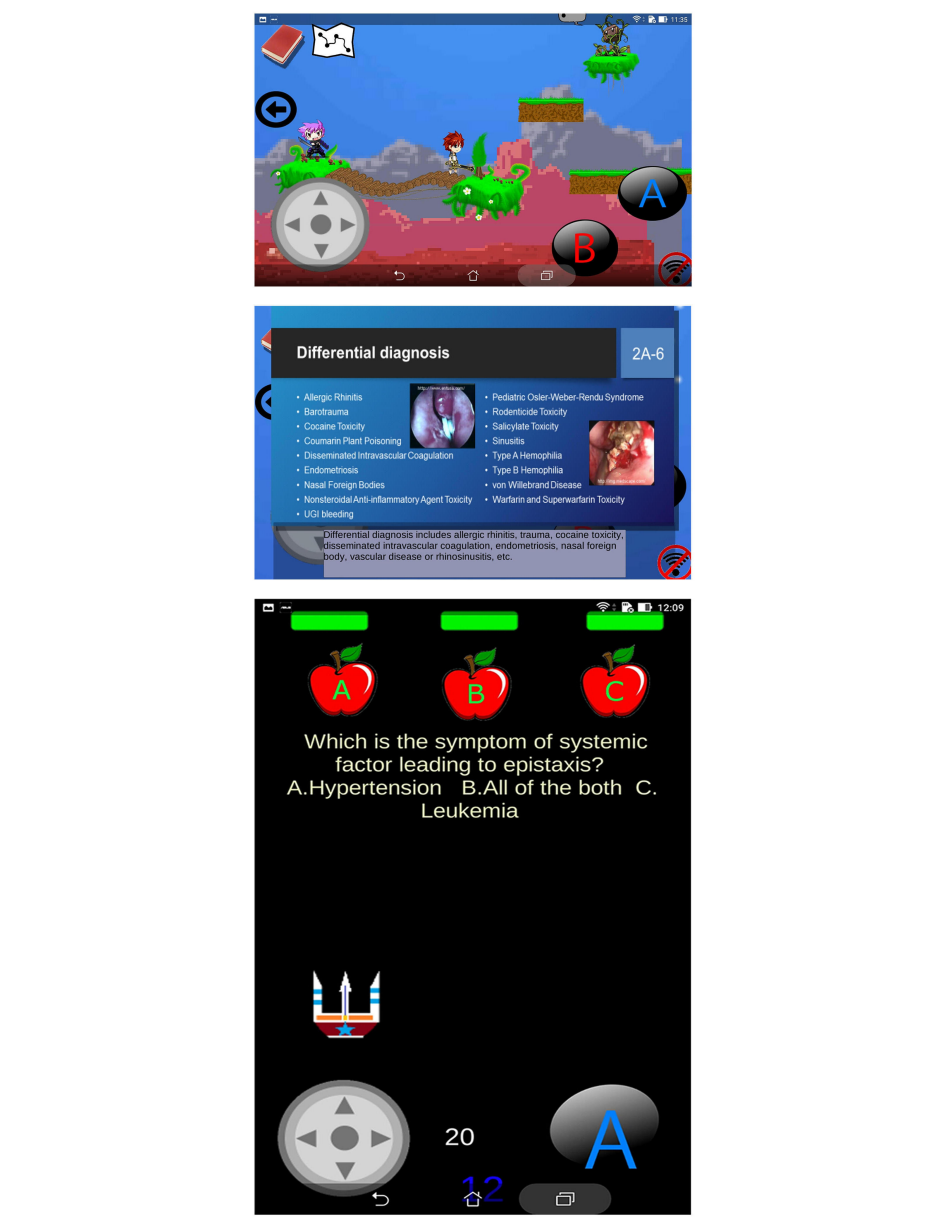
Screenshots of the interactive multimedia module. Learners arbitrarily operated a leading character to run, jump, and interact with other nonplayer characters (up) to procure instructional materials (middle). After a small session, learners need to complete small game-based quizzes (low).

Using the IM module, the learners could operate a leading character to search for and interact with other nonplayer characters to procure instructional materials, to review acquired instructional slides (maximal 80), and to win five small game-based quizzes (Figure 3). The instructional slides were briefly explained using scrolling text. The content for the novel IM module was derived from and corresponded to the textbook-based learning material of the conventional PPS module. Game-based quizzes with different contexts from the multiple-choice questions (MCQs) and multimedia situational tests (MSTs) provided small repetitive summaries of the emergent ORL-HNS disorders. The learners could use a learning map to assess their progress in each section or a bar chart to assess their progress overall.

#### The Conventional PPS Module

**Figure 4:**
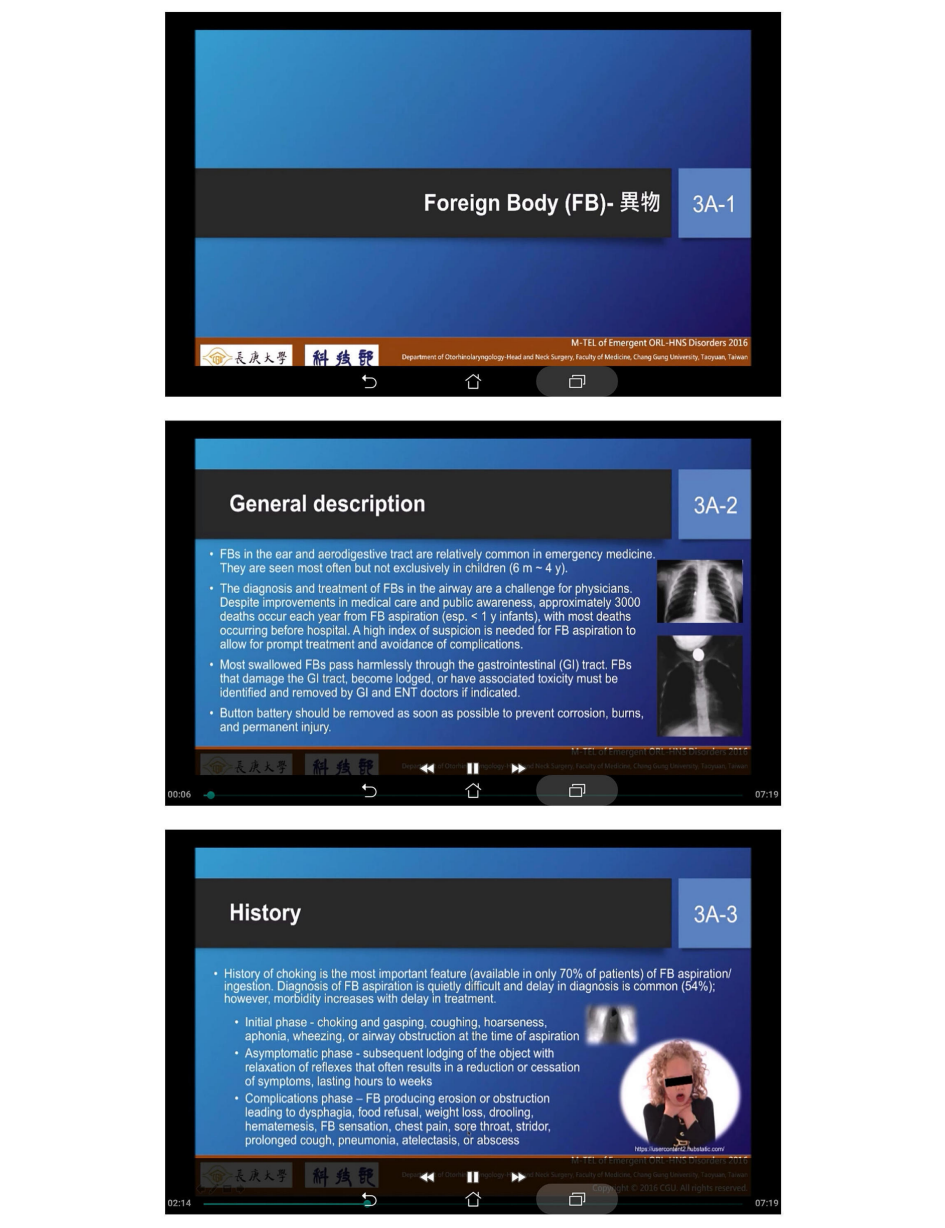
Screenshots of the PowerPoint Show module. Learners watched 10 visual-auditory text-image videos of emergent otorhinolaryngology-head and neck surgery (ORL-HNS) disorders. The instructional slides of this module were identical to those of the interactive multimedia module and arranged linearly.

In the PPS module, the learners chose and watched 10 visual-auditory text-image videos of emergent ORL-HNS disorders (a total of 80 min) by themselves. Video lectures were created by recording Microsoft PowerPoint presentations with audio narrations, timings, and ink gestures using Camtasia Studio software version 8 (TechSmith, Okemos, MI, USA). The learners were free to watch the videos at any time, and they could also rewind and fast-forward the videos as needed (Figure 4).

During the course, all of the participants arbitrarily reviewed 80 slides by themselves. The instructional content of the two learning modules was confirmed to be correlated and equivalent by 2 investigators from the study team (*r*=.91, *P*<.001, Spearman correlation test) using the Software Evaluation Checklist [25]. This checklist uses seven criteria (curriculum connections, age/grade appreciates, investment justification, lay-out, support materials, instructional content, and graphics/multimedia) with two (yes, no) Likert-type scales (a total of 28 questions). Qualitative feedback from the participants was obtained through feedback forms in the pilot study [23]. Major bug fixes were performed before the validation study.

The validation study was a prospective, parallel-controlled, randomized clinical trial assessing the impact of cognitive styles and learning modules using M-TEL on knowledge gain, competence gain, satisfaction, and learning experience.

### Selection of Participants

A total of 60 consecutive volunteers were recruited from a teaching clinic for the validation study from November 23, 2016 to July 5, 2017. All of the volunteers had at least a basic level of computer literacy, and they were shown the practical aspects of using tablets and apps. The inclusion criteria were as follows: (1) age >20 years; and (2) undergraduate medical students (clerkship). The exclusion criteria were: (1) previous ORL-HNS training; and (2) declining to participate.

### Methods of Measurement

There were four different face-to-face assessments. The cognitive style of the participants was assessed using the 25-item GEFT after enrollment [26]. The GEFT has a relatively high Spearman-Brown reliability coefficient of 0.82 [27]. On the basis of the number of correct answers given by the participants, the GEFT scores ranged from 0 (the most FD) to 18 (the most FI). We stratified the students into two subgroups: “classical FD” (GEFT score ≤12) and “classical FI” (GEFT score >12) [26].

Pretests including a 15-min 10-question standard MCQs to evaluate the students’ existing knowledge (range 0-100) and a 15-min 5-question MSTs to assess their existing competence (range 0-100) with regard to “emergent ORL-HNS disorders” were given to the students. Each textbook-based MCQ was designed to be answered within 90 seconds and was preselected according to the results of item analysis. The MSTs presented the learners with written descriptions of five scenarios with or without images/videos and asked them to select the appropriate responses from 5 MCQs for one emergent ORL-HNS disorder using the methodology described in a previous publication of key features approach [28]. The MST was developed to assess clinical reasoning competence [23]. After a 100-min learning course, the participants were again requested to answer a different set of MCQs and MSTs posttest. These assessments were comparable with respect to psychometric properties [26]. Two members of staff confirmed that these questions could be sufficiently answered after reviewing the instructional content of the M-TEL.

The students were then asked to complete a global satisfaction score (GSS) (range 0-10) questionnaire.

### Randomization and Blinding

**Figure 5:**
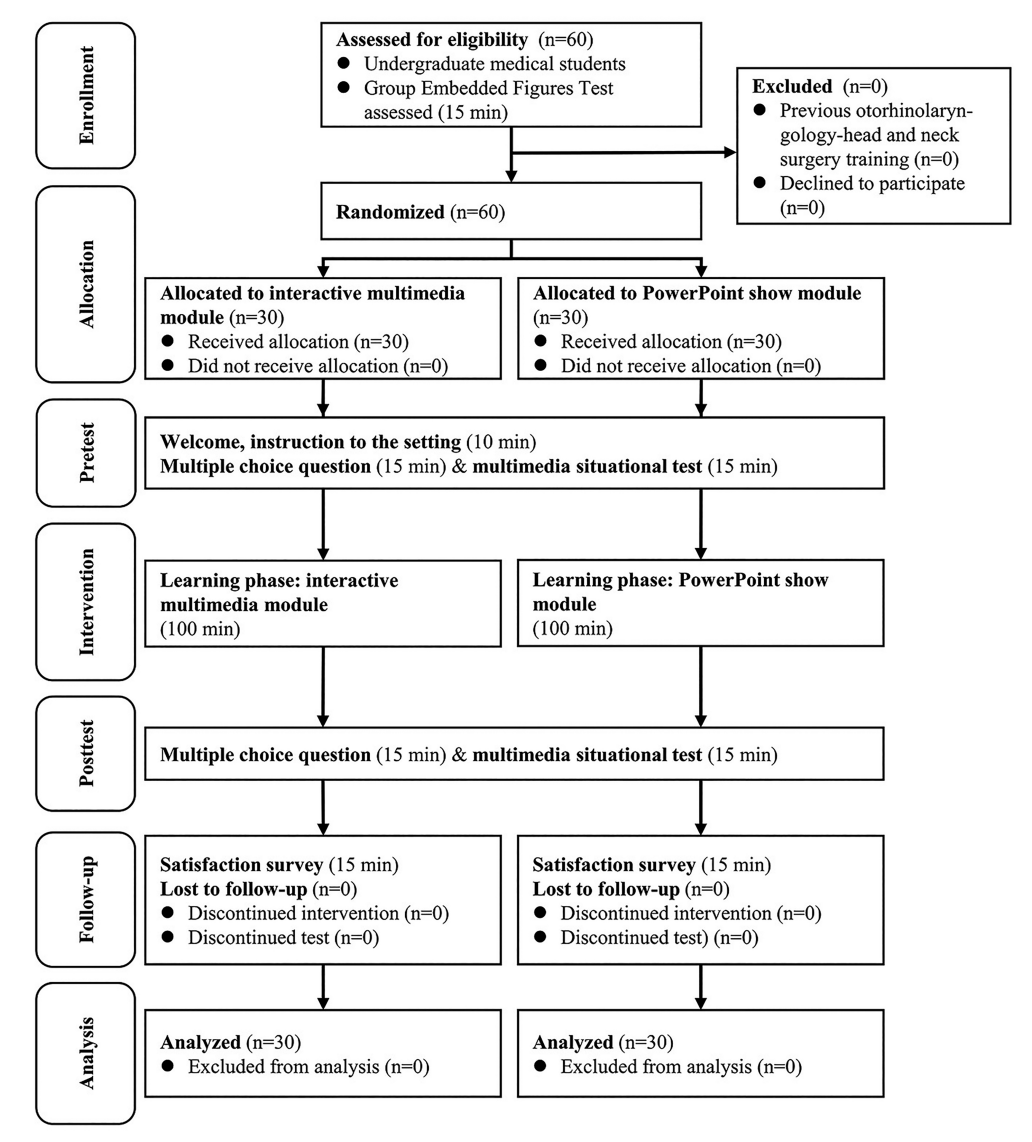
The Consolidated Standards of Reporting Trials flow diagram.

Blinding to the purpose of the study during recruitment was maintained to minimize preparation bias. After the participants had provided consent and completed the GEFT and pretests, we randomly assigned them (1:1) to the IM group and PPS group (Figure 5). A balanced design with regard to cognitive style, sex, and age was assured by the randomization procedure. Computer-generated lists of random numbers were created using the Random Number Generator in IBM SPSS software version 23 (IBM, Armonk, NY, USA) for the allocation of the students, who were stratified by center with a 1:1 allocation using a fixed block size of 6 in both parallel subgroups. The allocation sequence was concealed before implementation of the M-TEL module, and the module adhered to our computer-generated randomization protocol.

### Intervention

After randomization, the participants were unblinded and used fully automated courseware containing IM or PPS module on a 7-inch tablet in an ordinary office environment for 100 min. Before using the courseware, the functionality of the tablet was explained to the participants. The IM group participated in a parkour course to find and read the instructional materials and played small quiz games that were different from the MCQ and MST questions. The students in the PPS group used an app to read and listen to instructional materials in 10 linear-designed sessions. After completing the brief sessions, the IM and PPS learners could review simple slides of the instructional materials.

### Outcome Measures

The primary outcome measure was the percentage change in MCQ score (ie, “knowledge gain”) after the M-TEL. Other outcomes were the percentage changes in MST (ie, “competence gain”) and GSS.

### Sample Size

A priori sample size was estimated using primary outcome effects (percentage change in MCQ score) based on a pilot study (IM module: 43% [SD 18%]; PPS module: 35% [SD 21%]). A two-tailed Wilcoxon signed-rank test to calculate the sample size of 26 in each group (normal parent distribution; calculated effect size: 0.41; type I error: 0.05; power: 80%). Assuming a 10% dropout rate to fulfill the criteria of intention-to-treat analysis, we needed at least 29 participants in each group. Accordingly, we decided to enroll a total of 60 students to show the difference in percentage change in MCQ score.

### Statistical Analysis

Because the primary outcome measure (percentage change in MCQ score) was not normally distributed according to the D’Agostino-Pearson omnibus normality test, percentage changes ([after value-before value]/[before value] × 100) in MCQ, MST, and GSS; and AttrakDiff2 scores were compared between groups using the Wilcoxon signed-rank test, Mann-Whitney *U* test, or Kruskal-Wallis test as appropriate. Categorical variables were analyzed using Fisher exact test, and the Spearman correlation test was used to analyze the relationship between variables of interest. Friedman test (two-way nonparametric analysis of variance) was used to compare the effect of multiple levels of two factors [29]. All tests were two-tailed, and statistical significance was established at *P*<.05. Statistical analyses were performed using G*Power 3.1.9.2 software (Heinrich-Heine University, Dusseldorf, Germany), Statistical Package for the Social Sciences for Windows version 23.0 (SPSS Inc., Chicago, IL, USA), and GraphPad Prism for Windows version 7.0 (GraphPad Software Inc., San Diego, CA, USA).

## Results

### Study Participants

A total of 60 undergraduate medical students were screened, all of whom (median age 23 years, range 22-26 years; 36 males, 60% and 24 females , 40%) were randomized 1:1 to the IM group or PPS group as shown in the Consolidated Standards of Reporting Trials flow diagram (Figure 3; Multimedia Appendix 1 [30]). Table 1 summarizes the variables of interest for the overall study cohort. There were no significant differences in age, sex, cognitive style, MCQ, or MST scores between the two groups at baseline. All of the participants (100%, 60/60) received the intended intervention after randomization, and there was no deviation from the study protocol.

### Primary and Secondary Outcomes

Overall, all of the participants showed significant improvements in MCQ score (*P*<.001) and MST score (*P*=.006) after 100 min of e-learning (Table 1). The median percentage changes in MCQ and MST scores were 50% (interquartile range 17%-80%, *P*<.001) and 13% (interquartile range 0%-33%, *P*=.006), respectively. The M-TEL positively impacted the GSS (*P*<.001). The PPS group had significant improvements in knowledge (*P*<.001) and competence (*P*=.001), whereas the IM group had a significant improvement in knowledge (*P*<.001) but not in competence (*P*=.53). There were no significant differences in the percentage changes in the MCQ or MST score between the two groups (*P*=.42 and *P*=.16, respectively). Notably, the IM group had a significantly higher GSS compared with the PPS group (*P*=.01).

### Differences in Outcomes Between the Classical Field-Dependent and Field-Independent Learners

Using the original definition of FD and FI defined by Witkin [29], 5 (8%) participants had the classical FD cognitive style and 55 (92%) had the classical FI cognitive style. Table 2 summarizes comparisons of the variables of interest between the classical FD and FI learners. There were no significant differences in age, sex, M-TEL module, or MCQ or MST scores between the classical FD and FI groups at baseline. After 100 min of M-TEL, increases in MCQ and MST scores in the classical FD learners did not reach statistical significance (*P*=.14 and *P*=.85, respectively), whereas they were significantly increased in the classical FI learners (*P*<.001 and *P*=.003, respectively). However, the percentage changes in MCQ and MST scores were not significantly different between the classical FD and FI groups (*P*=.90 and *P*=.68, respectively). Even though the differences were not statistically significant, the classical FD learners had a lower GSS than the classical FI learners.

**Table 1:** Demographics, cognitive style, learning outcomes, satisfaction, and experience.

Variables		Overall, N=60	Interactive multimedia group, N=30	PowerPoint show group, N=30	*P* value^a^
**Demographics**					
	Age in years, median (IQR^b^)	23 (23-24)	23 (23-24)	23 (23-24)	.21
	Male sex, n (%)	36 (60)	20 (67)	16 (53)	.43
**Cognitive style**					
	Group embedded figures test score, median (IQR)	17 (15-18)	18 (15-18)	17 (16-18)	.78
	Field-dependence, n (%)	5 (8)	3 (10)	2 (7)	>.99
**Learning outcomes**					
	Multiple-choice questions-before, median (IQR)	40 (40-50)^c^	40 (40-60)^c^	45 (30-50)^c^	.47
	Multiple-choice questions-after, median (IQR)	70 (60-80)^c^	70 (58-70)^c^	70 (60-80)^c^	.72
	Percentage change in multiple-choice questions, median (IQR)	50 (17-80)^d^	40 (13-76)^d^	60 (20-100)^d^	.42
	Multimedia situational test-before, median (IQR)	80 (60-80)^c^	80 (60-80)	80 (60-80)^c^	.84
	Multimedia situational test-after, median (IQR)	80 (80-100)^c^	80 (60-80)	80 (80-100)^c^	.003
	Percentage change in multimedia situational test, median (IQR)	13 (0-33)^d^	0 (−21 to 38)	25 (0-33)^d^	.16
**Learning satisfaction**					
	Global satisfaction score, median (IQR)	7 (5-9)^d^	8 (6-9)^d^	6 (4-7)	.01

^a^Mann-Whiney *U* test (continuous variables) or Fisher exact test (categorical variables)

^b^IQR: interquartile range.

^c^*P*<.05, before versus after, Wilcoxon signed-rank test (two-tailed).

^d^*P*<.05, compared with a neutral value (“0” for multiple-choice questions and multimedia situational test, or “5” for “global satisfaction score”), Wilcoxon signed-rank test (two-tailed).

**Table 2:** Comparisons of demographics, learning model, outcomes, satisfaction, and experience between classical cognitive styles.

Variables		Classical field-dependent, N=5	Classical field-independent, N=55	*P* value^a^
**Demographics**				
	Age in years, median (IQR^b^)	23 (22-24)	23 (23-24)	.45
	Male sex, n (%)	2 (40)	34 (62)	.38
	Group embedded figures test score, median (IQR)	9 (4-12)	18 (17-18)	<.001
**Learning model**				
	Interactive multimedia, n (%)	3 (60)	28 (51)	>.99
**Learning outcomes**				
	Multiple-choice questions-before, median (IQR)	40 (25-60)	40 (40-50)^c^	.53
	Multiple-choice questions-after, median (IQR)	60 (50-80)	70 (60-80)^c^	.70
	Percentage change in multiple-choice question, median (IQR)	67 (−7 to 200)	50 (17-80)^d^	.90
	Multimedia situational test-before, median (IQR)	80 (50-100)	80 (60-80)^c^	.63
	Multimedia situational test-after, median (IQR)	80 (70-90)	80 (80-100)^c^	.92
	Percentage change in multimedia situational test, median (IQR)	0 (−30 to 92)	25 (0-33)^d^	.68
**Learning satisfaction**				
	Global satisfaction score, median (IQR)	6 (4-7)	7 (5-9)^d^	.25

^a^Mann-Whiney *U* test (continuous variables) or Fisher exact test (categorical variables).

^b^IQR: interquartile range.

^c^*P*<.05, before versus after, Wilcoxon signed-rank test (two-tailed).

^d^*P*<.05, compared with a neutral value (“0” for multiple-choice question and multimedia situational test, or “5” for “global satisfaction score”), Wilcoxon signed-rank test (two-tailed).

### Post Hoc Analysis

In this study, most of the participants were categorized as classical FI learners, and we were unable to determine which FI learners were more suitable for M-TEL using classical classification [26]. El-Banna proposed that a field-intermediate (FINT) category exists between FD and FI categories [31]. Accordingly, we adopted this modified classification of cognitive style (FD: <mean GEFT score − standard deviation [SD] × 0.25; FINT: ≥mean GEFT score − SD × 0.25 and ≤mean GEFT score + SD × 0.25; FI: >mean GEFT score + SD × 0.25); thereby resulting in three modified categories : FD: <16, n=15 (25%); FINT: ≥16 and ≤17, n=17 (28%0; FI: >17, n=28 (47%). Table 3 illustrates comparisons of these modified FD, FINT, and FI groups using modern classification. There were no significant differences in age, sex, M-TEL module, or MCQ or MST scores among the modified FD, FINT, and FI groups at baseline. Modified FI was independent of the M-TEL module and learning outcomes (all *P*>.05). Increases in MCQ and MST were significant in all three of these cognitive style groups. Although the differences in knowledge and competence gains were not statistically significant among the modified cognitive styles, the FINT group had a significantly higher satisfaction with M-TEL than the modified FD group regardless of which M-TEL module they used. The modified FINT learners had a significantly positive attitude toward M-TEL in terms of GSS, whereas the modified FD learners had the lowest GSS. Furthermore, the FINT learners using the IM module had a significantly higher GSS than those using the PPS module (*P*=.005). The modified FI learners had a positive attitude toward M-TEL in terms of GSS, and a significantly higher GSS when using the IM module compared with the PPS module (*P*=.02). We further compared the effect of modified cognitive style and M-TEL module on outcomes using Friedman test (Table 4). Both modified cognitive style and M-TEL module had significant effects on percentage changes in MCQ score and GSS. The M-TEL module had significant effects on MST score, whereas modified cognitive style did not have any significant effect.

**Table 3:** Comparisons of demographics, learning models, outcomes, satisfaction, and experience among modified cognitive styles.

Variables		Modified field-dependent, N=15	Modified field-intermediate, N=17	Modified field-independent, N=28	*P* value^a^
**Demographics**					
	Age in years, median (IQR^b^)	23 (22-24)	23 (23-24)	23 (23-24)	.74
	Male sex, n (%)	8 (53)	12 (71)	16 (57)	.56
**Cognitive style**					
	Group embedded figures test score, median (IQR)	14 (12-15)	17 (17-17)	18	<.001
**Learning module**					
	Interactive multimedia, n (%)	8 (53)	7 (41)	15 (54)	.69
**Learning outcomes**					
	Multiple-choice questions-before, median (IQR)	50 (30-60)^c^	40 (40-50)^c^	40 (40-50)^c^	.47
	Multiple-choice questions-after, median (IQR)	70 (60-80)^c^	70 (65-80)^c^	70 (53-70)^c^	.48
	Percentage change in multiple-choice question, median (IQR)	40 (17-100)^d^	75 (33-100)^d^	45 (15-75)^d^	.34
	Multimedia situational test-before, median (IQR)	80 (60-80)^c^	80 (60-80)^c^	70 (60-80)^c^	.74
	Multimedia situational test-after, median (IQR)	80 (80-80)^c^	80 (70-100)^c^	80 (63-95)^c^	.83
	Percentage change in multimedia situational test, median (IQR)	25 (0-33)^d^	25 (0-29)^d^	0.0 (0-37)^d^	.82
**Learning satisfaction**					
	Global satisfaction score, median (IQR)	6 (3-7)	8 (7-10)^d^	7 (5-8)^d^	.02

^a^Mann-Whiney *U* test (continuous variables) or Fisher exact test (categorical variables).

^b^IQR: interquartile range.

^c^*P*<.05, before versus after, Wilcoxon signed-rank test (two-tailed).

^d^*P*<.05, compared with a neutral value (“0” for multiple-choice question and multimedia situational test, or “5” for “global satisfaction score”), Wilcoxon signed-rank test (two-tailed).

**Table 4:** Comparisons of the effect of modified cognitive style and module of mobile technology in electronic learning on outcomes.

Outcomes^a^		Test statistics	Standard error	Standard test statistics	*P* value	Adjusted *P* value
**Percentage change in multiple-choice question**						
	Modified cognitive style–learning module	−0.53	0.18	−2.92	.003	.01
	Modified cognitive style–percentage change	0.71	0.18	−3.88	<.001	<.001
	Learning module–percentage change	−1.24	0.18	−6.80	<.001	<.001
**Percentage change in multimedia situational test**						
	Modified cognitive style–learning module	−0.57	0.18	−3.10	.002	.006
	Modified cognitive style–percentage change	0.11	0.18	0.59	.55	>.99
	Learning module–percentage change	−0.46	0.18	2.51	.012	.04
**Global satisfaction score**				−		
	Modified cognitive style–learning module	−0.52	0.18	−2.83	.005	.01
	Modified cognitive style–global satisfaction score	−1.19	0.18	−6.53	<.001	<.001
	Learning module–global satisfaction score	−1.71	0.18	−9.36	<.001	<.001

^a^Friedman’s two way analysis of variance test.

## Discussion

### Principal Findings

The main findings of this study are that M-TEL outside the classroom can help undergraduate medical students to strengthen their knowledge and competence of emergent ORL-HNS disorders, and to provide an enjoyable learning experience overall. In addition, our findings suggest that millennials can significantly gain knowledge rather than reinforce competence using an IM module. Despite the similar efficacy of both modules, the students preferred the IM module to the PPS module because of it being more efficient and enjoyable to use. Although the classical classification of cognitive style [26] did not seem to be associated with learning preference or outcomes, the modified FINT learners had the highest knowledge gain and satisfaction with M-TEL (especially the IM module) compared with the modified FD and FI learners [32]. With further controlling for the modified cognitive style, the PPS module enhanced competence compared with the IM module.

### Limitations

There are several limitations to this randomized controlled trial. First, this study was quasi-experimental because of the lack of probability sampling. Even though we selected individuals based on their availability to the investigators, the sample size was representative of the target population (>50% were classmates). Second, the study used different posttest questions to measure learning outcomes, and the interaction between taking a pretest and the intervention itself may threaten the external validity. A design which does not use a pretest would have been preferable [33]. Third, we did not survey learning preferences, cognitive load, or self-direction in this short-term study. These factors have been reported to effect learning outcomes in modern medical curricula [16,34], and they should be closely monitored when students use this type of M-TEL app.

### Comparison With Prior Work

As mentioned above, both the IM and PPS modules used the same textbook-based learning material. However, the IM module applied game-design elements and game principles in nongame contexts (gamification) [35]. The gamification of medical education is a rapidly growing field [32,36], and it has been shown to have the potential to improve knowledge [37], and increase motivation and engagement [38]. Our results demonstrate that M-TEL for medical education can facilitate the learning of complex topics with promising results in terms of gains in knowledge, competence, and satisfaction compared with other forms of e-learning [39-41]. However, the learners using the gamified IM model still struggled with performance in MSTs. Therefore, M-TEL may not be an approach that is suitable for all.

Teaching and learning processes are thought to be affected by various cognitive variables. If medical students receive training, which has been designed according to their individual needs, they may develop a sense of competence and positive self-perception [42]. Previous studies have shown that FI learners have higher levels of achievement and better problem-solving ability [43,44]. In this study, relatively few of our undergraduate medical students were classical FD learners, they did not seem to significantly gain knowledge or competence after the 100-min M-TEL module, and they were neutrally satisfied with this learning method. This may be due to the small sample size or suboptimal classification of cognitive style. Therefore, we applied the modified classification of cognitive style that has been used for gifted students [45] and classified our participants accordingly. Using this modified classification, we found that all of the participants significantly gained knowledge and competence after the M-TEL course regardless of cognitive style. In this short-term M-TEL course, the FINT learners had the significantly highest knowledge gain and satisfaction regardless of which M-TEL module they used compared with the modified FD and FI learners, who needed a more specific design of instructional material. For example, the FD learners needed an easy-to-use and follow style of M-TEL, whereas the FI learners wanted a more vigorous style of M-TEL, including engaging quiz games [23].

Medical teachers in Taiwan have traditionally assumed that medical students can automatically adapt to the instructional modality and material by themselves to learn a topic, regardless of whether or not they are effective. Although we previously postulated that both learning module and cognitive style could affect competence gain for UME, we found the undergraduate medical students using the PPS learning module had significantly higher improvements in competence, whereas there was no change in competence in the IM learners. Even though the IM module was more attractive and satisfactory than the PPS module, more integrated formats of instructional material were needed to reduce extraneous cognitive load to facilitate problem-solving performance [44]. Bertini et al [46] concluded that FI learners can effectively transfer tasks when they must be transferred to a novel situation and that they can identify the important aspects of ambiguous or disorganized information. However, in this study, modified cognitive styles did not significantly affect the MST outcomes whereas the learning module did (using Friedman test). Even though most of our participants had the FI cognitive style making them better suited to game-based tasks, but insensitive to situational cues, and the minority had the FD cognitive style resulting in divided attention and increased cognitive load in game-based processing, all of them had a similar posttest MST performance. Nevertheless, the high overall baseline MST score suggests that the study participants were previously equipped with an understanding of the clinical reasoning process [47] and that this helped them to predict the features of clinical situations, to determine the appropriate course of action, and to choose correct solutions in the MST. Even though they were novices in ORL-HNS, the high baseline scores created a buffer to achieve a significant increase in MST performance.

Since learners can start and stop M-TEL at any time or place of their choosing [48], this learning modality may be superior to traditional classroom lectures with regard to self-directed effort management and organized study, and allow them to achieve deep understanding by repeatedly reviewing the instructional materials. Because learning preference and satisfaction allow students to learn outside the classroom, cognitive style should be taken into consideration to design curricula to suit the style of the individual undergraduate medical student, thereby enhancing the effectiveness of M-TEL [49].

### Conclusions

M-TEL using conventional PPS and novel IM modules seems to be an effective method to teach emergent ORL-HNS disorders to undergraduate medical students. The PPS module represented a formal, serious learning modality, whereas the IM module represented a satisfactory, enjoyable way for the millennial students to learn. Cognitive style and M-TEL module significantly affected knowledge gain and satisfaction, and the modified FINT learners had the highest gains in knowledge and satisfaction when using the IM module. These findings support the development of M-TEL, including various learning modules for undergraduate medical students with different cognitive styles.

## Appendix

Multimedia Appendix 1 CONSORT-EHEALTH checklist V 1.6.1.

## Abbreviations

FD: field-dependent
FI: field-independent
FINT: field-intermediate
GEFT: group embedded figure test
GSS: global satisfaction score
IM: interactive multimedia
IQR: interquartile range
MCQ: multiple-choice questions
MST: multimedia situational test
M-TEL: mobile technology in e-learning
ORL-HNS: otorhinolaryngology-head and neck surgery
PPS: PowerPoint show
UME: undergraduate medical education
